# Digital Health Tools Can Support Patient Access to Culturally and Linguistically Competent Behavioral Health Treatment

**DOI:** 10.2196/51799

**Published:** 2023-08-25

**Authors:** Mitchell Berger

**Affiliations:** 1 US Department of Health and Human Services Rockville, MD United States

**Keywords:** African American, women, depression, telemedicine, mobile health, mHealth, mobile apps, digital health, mental health, gender minority, mobile technology, mobile phone

As significant investments of funding and time go toward digital health tools, it seems to be a mantra among many in the behavioral health space that “if you build it, they will come” or, less colloquially, that digital technologies, once developed and approved for use, will be supported and rapidly adopted both by patients and providers. It, however, remains somewhat unclear just how much enthusiasm for digital health tools really exists, who will use them, and how they will fit among other traditional and innovative therapeutic modalities. Thus, it was interesting and important to read McCall et al’s [1] research and conclusion that “Black American women, in general, have favorable views toward seeking mental health services and are comfortable with the use of mobile technology to receive support for managing depression.”

Their findings dovetail with other research showing that, while digital therapies are likely to be effective, equity, privacy, ethics, and reimbursement considerations must be part of the discussion as these therapies are being developed [2,3]. The Substance Abuse and Mental Health Services Administration in a recent issue brief explored regulatory, research, and equity considerations associated with digital therapeutics, noting that future therapies “must be designed and implemented to account for differences in health and digital literacy and to be culturally and linguistically appropriate, adaptable to variable service settings, and affordable and accessible for all users” [2]. Research like that of McCall et al [1] helps providers, policy makers, and patients better understand how patients may perceive the benefits and drawbacks of digital health tools. As McCall et al [1] acknowledge, their research may not necessarily be broadly applicable to other populations, and additional understanding of how digital therapeutics are perceived across income levels, geographic areas, race and ethnicity, sexual orientation, and other demographic groups would be helpful. Ideally, digital health tools will complement broader efforts to promote equity in behavioral health screening, access, prevention, and treatment.

In addition to concerns about disparities and discrimination, which McCall et al [1] reference in their article, products intended for use (or even likely to be used) by minors also will need special consideration [4]. Additionally, it is worth exploring the use of digital health tools to support the behavioral health of geriatric populations [5].

With more digital tools arriving amid a shortage of behavioral health providers, digital tools have the potential, as during COVID-19, to help reach additional populations and support a busy and challenged provider workforce. Working together, we can ensure that post–COVID-19 digital approaches are grounded in an understanding of the needs, values, and perspectives of patients and behavioral health providers who will use and hopefully benefit from these additional tools.
